# Hollow Filaments from Coaxial Dry–Jet Wet Spinning of a Cellulose Solution in an Ionic Liquid: Wet-Strength and Water Interactions

**DOI:** 10.1021/acs.biomac.3c00984

**Published:** 2023-12-12

**Authors:** Shiying Zhang, Guillermo Reyes, Alexey Khakalo, Orlando J. Rojas

**Affiliations:** † Department of Bioproducts and Biosystems, School of Chemical Engineering, Aalto University, FI-02150 Espoo, Finland; ‡ VTT Technical Research Center of Finland, Fl-02150 Espoo, Finland; § Bioproducts Institute, Department of Chemical & Biological Engineering, Department of Chemistry and Department of Wood Science, The University of British Columbia, 2360 East Mall, Vancouver, BC V6T 1Z3, Canada

## Abstract

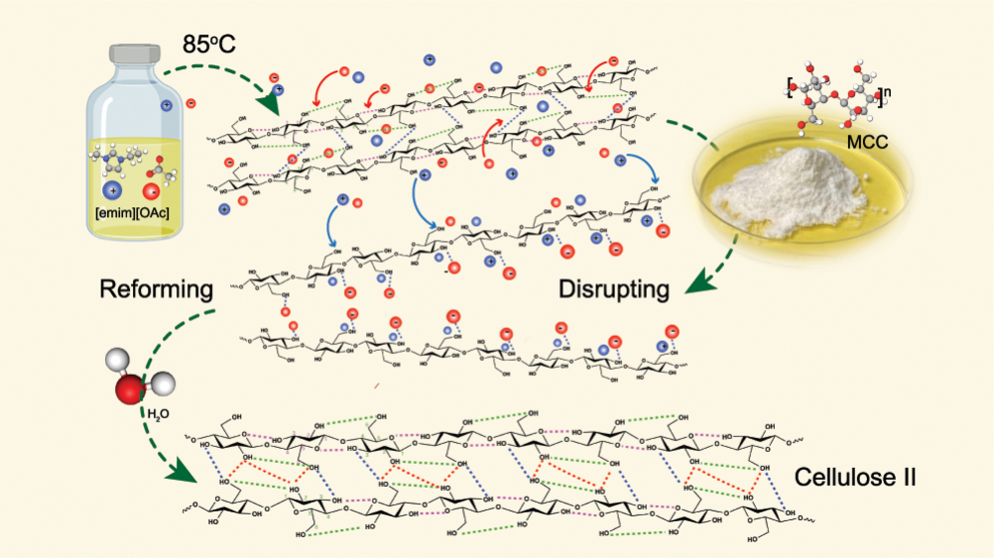

Hollow tubing and tubular filaments are highly relevant to membrane technologies, vascular tissue engineering, and others. In this context, we introduce hollow filaments (HF) produced through coaxial dry–jet wet spinning of cellulose dissolved in an ionic liquid ([emim][OAc]). The HF, developed upon regeneration in water (23 °C), displays superior mechanical performance (168 MPa stiffness and 60% stretchability) compared to biobased counterparts, such as those based on collagen. The results are rationalized by the effects of crystallinity, polymer orientation, and other factors associated with rheology, thermal stability, and dynamic vapor sorption. The tensile strength and strain of the HF (dry and wet) are enhanced by drying and wetting cycles (water vapor sorption and desorption experiments). Overall, we unveil the role of water molecules in the wet performance of HF produced by cellulose regeneration from [emim][OAc], which offers a basis for selecting suitable applications.

## Introduction

Membrane technologies have gained significant prominence in various scientific and industrial applications, including but not limited to water treatment, gas separation, and biomedical engineering.^1^ The versatility of membranes lies in their ability to selectively separate, filter, and transport specific components, rendering them indispensable tools in numerous fields. Within the realm of membrane technology, hollow filaments (HF) have emerged as an appealing configuration due to their self-supporting properties and high surface-area-to-volume ratio.^2^ These characteristics make HF well-suited for various applications, ranging from filtration and cell culturing to tissue and scaffold engineering.^1−5^ Unfortunately, current commercial HF systems are typically produced from oil-based sources, such as poly(vinylidene fluoride), polyacrylonitrile, polysulfone, poly(ether sulfone), and poly(tetrafluoroethylene), which rely heavily on toxic solvents such as *N*-methylpyrrolidone, *N*,*N*-dimethylacetamide, and *N*,*N*-dimethylformamide.^2^ Moreover, membrane production industries generate wastewater, and recycling is difficult and not economical.^2,6^ To tackle this impact and introduce the possibility of establishing an environmentally sound option, green solvents and renewable natural resources are suggested as attractive precursors to fabricate HF with low operational impact.^2,6,7^ In this context, cellulose, a naturally occurring polymer, is positioned as a suitable alternative to petroleum-based resources, providing the opportunity for advancing material development due to its high biocompatibility, thermal and chemical stability, and biodegradability.^7^ High interest exists on the need for developing a durable cellulose HF capable of performing under harsh conditions (high moisture and temperatures), e.g., to remedy the structural instability and fragility commonly associated with their biobased counterparts.^4^

It is noteworthy that the pursuit of environmentally sustainable HF extends beyond the selection of raw material precursors. This necessitates a comprehensive consideration of technical, economic, and environmental aspects, particularly in terms of the fabrication techniques employed. Among the array of conventional HF fabrication methods, which encompass nonsolvent-induced phase separation (NIPS), thermally induced phase separation, melt-extrusion, and track-etching, the NIPS technique is the most promising for industrial-scale production. This preference is attributed to its simplicity and the outstanding performance of the resultant membranes.^6^ To implement the NIPS method, three essential components are required: a polymer to define the membrane properties, a solvent to dissolve the polymer, and a nonsolvent to control phase inversion phenomena.^2,6^ Cellulose solution would be a suitable precursor to synthesize environmentally friendly HF because of its readily regeneration ability in water (antisolvent).^7−9^ However, the nature of cellulose’s molecular structure and its inherent recalcitrance impose a challenge on reactivity and dissolution.^7−9^ Hence, there is a demand for novel processing media, such as green solvents, which can offer improved energy efficiency and are amenable to recycling and reusability.^6,7^

Among the available cellulose solvents, nonderivatizing ionic liquids (ILs) have been known for their great prospects, as ascertained by developments reported during the last 15 years.^10−12^ Specifically, imidazolium-based ILs, incorporating an acetate anion, such as 1-ethyl-3-methylimidazolium acetate ([emim][OAc]), have demonstrated notable biomass dissolution capacity.^13^ Furthermore, the recycling of [emim][OAc] used in biomass dissolution can achieve a recovery rate exceeding 85%, with no observed structural alterations.^7,13^ Additionally, compared to the well-established NMMO process, imidazolium-based ILs exhibit a favorable environmental performance in terms of abiotic resource depletion, reduction of volatile organic compound emissions, and ecotoxicity.^14−16^ The use of ILs has promoted the development of functional cellulose filaments via a one-step physical NIPS method through a dry–jet wet spinning configuration.^7,17,18^

Responding to these needs, herein, we introduce the coaxial dry–wet spinning of cellulose dissolved in [emim][OAc] as an effective and suitable approach to achieve regenerated cellulose HF. We deepen the molecular interactions between regenerated cellulose and water to comprehend the extended mechanical performance of the obtained HF. This advancement holds promising potential to meet the needs of membrane industries, serving as a framework for further research related to functional materials.

## Materials and Methods

### Materials

Microcrystalline cellulose (MCC, Avicel PH-101, powder form) obtained from cotton linters and with a particle size of 50 μm was purchased from Sigma-Aldrich. MCC was selected for its high purity (>99%) and degree of crystallinity (65–93%).^19^ IL [emim][OAc], with a purity of ≥98% and a molecular weight of 170.2 g/mol, was purchased from Proionic and stored under inert conditions until use. Deionized water was employed as a bore fluid and coagulant.

### Cellulose Dissolution

MCC and [emim][OAc] were vacuum-dried in an oven (Thermo Scientific, VT 6025, Fisher Scientific Oy, Vantaa, Finland) at 70 °C, 200 mbar for 24 h to remove any moisture, given its detrimental effect on dissolution in ILs.^20^ MCC dissolution in [emim][OAc] was carried out using a vertical kneader system^18^ (85 °C, 30 rpm, 45 mbar, 3 h). The obtained [emim][OAc]-MCC solutions were filtrated to remove undissolved residues with the help of a hydraulic filter press (200 bar, 85 °C) equipped with a metal mesh (5 mm pore size, Gebr. Kufferath AG, Germany).

### Dry–Jet Wet Spinning

The coaxial dry–jet wet spinning technique was used to produce HF (Figure S1a). The outer and inner diameters of the coaxial spinning needles (Rame-Hart Instrument Co., Succasunna) corresponded to gauge 14 (1.6 mm) and gauge 21 (0.508 mm). The [emim][OAc]-MCC solution (Figure S1b) was filled into a 50 mL stainless steel syringe (Chemyx Inc.) and kept throughout the spinning process at 80 °C by a heating adapter (Syribge Heater, New Era Pump System Inc.). The bore fluid (deionized water) was filled into a 60 mL plastic syringe individually connected to a syringe pump (Chemyx Inc., Fusion 6000 high-pressure model). The extrusion velocity of the cellulose solution (shell) was fixed at the same speed as that of the bore fluid. Cellulose solutions (concentrations of 6 and 8 wt %) were spun at 6 mL/min, while those at 12 wt % were processed at 6, 10, and 12 mL/min. The corresponding HF are hereafter referred to as HF-*n*–*m*, where *n* and *m* correspond to the cellulose wt % concentration and extrusion speed (mL/min). The vertical distance between the spinning needle and the coagulation bath (air gap) was kept at 2 cm (HF-6–6, HF-8–6, and HF-12–6), but it was increased to 3 cm for HF-12–10 and HF-12–12, promoting additional gravity elongation stress.^21^ Deionized water (at room temperature, 23 °C) was employed as the nonsolvent in the coagulation bath for cellulose regeneration. The coagulated HF was washed three times in hot water (60 °C) to remove excess IL and subsequently dried at 23 °C at 30 or 50% relative humidity (RH) and under tension (using fixed filament ends; Figure S1c).

### Rheological and Dry/Wet Strength

The viscoelastic and flow properties of cellulose dissolutions (spinning dopes) were accessed by using an Anton Paar MCR302 stress-controlled rheometer. The impact of temperature and material concentration was studied with measurements using a parallel, aluminum cone (1°, 25 mm in diameter) and a Peltier heating plate. The shear viscosity was investigated following a logarithmic ramp (100–1 s^–1^, 21 points) and using shear rates from 0.1 to 100 s^–1^ (1–1000 s^–1^ for visualization using an optical camera with a polarized light filter). A strain of 0.1% was selected from the linear viscoelastic region on the amplitude sweep test for oscillatory tests (Figure S2). Master curves (Figure S3) were predicted at a higher angular frequency (>100/rad). The Cox–Merz rule was confirmed by fitting complex viscosity with shear viscosity. The temperature-dependent viscosity (shear rate of 10 s^–1^, Figure S4) was determined between 25 and 95 °C.

The mechanical strength of the HF was examined via tensile tests using a universal tensile device (Instron 4204) with a 100-N load cell operated at a 1 mm/min speed. The wet strength of the HF was obtained by prewetting samples with deionized water for 24 h (Figure S6). Six replicas of each sample were analyzed, following the ASTM D3822/D3822 M standard. The tensile strength and elastic modulus of the HF are calculated according to eqs S3 and S4, respectively.

### Chemical and Morphological Features

The surface topography and cross-sectional morphology of the HFs were investigated by scanning electron microscopy (SEM, Phenom Pure G5, Phenom-World, Netherlands). For this purpose, the given HF was cryo-fractured in liquid nitrogen and subsequently coated with a thin layer of an 80/20 gold and palladium (Sputter Q150R plus, Quorum, UK). The molecular fingerprints of MCC and HF were analyzed by FTIR-ATR (Spectrum Two FTIR Spectrometer, PerkinElmer, UK). Each FTIR measurement was conducted using 40 scans at 2 cm^–1^ resolution, and air background was considered in the analysis.

### Structural Features

The fiber orientation factor, crystallinity index, and lateral crystallite size of MCC and the HF were examined by a bench beamline WASX device (Xenoxs, Xeuss 3.0, UK). The generator operated at 45 kV and 200 mA with Cu Ka radiation (1.54056 Å). Three different spots were examined for each sample. Background corrections accounting for the sample holder and air were made by subtracting the corresponding signal in the absence of the sample. Fityk software (fityk-1.3.1) was employed for carrying deconvolution. MCC and HF’s empirical crystallinity indices (*CI*) were calculated using the empirical Segal method (eq 1)^22^
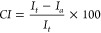
1where *I*_*t*_ is the intensity of the crystalline peak (21.9°/020) for cellulose II and (22.55°/200) cellulose I, while *I*_*a*_ is the respective amorphous peak located at 16.7 and 17.2° for cellulose II and cellulose I. Note that the definition of cellulose crystallinity can be somewhat problematic since it varies depending on the measurement and fitting method used.^23^ For cellulose characterization, one must be aware of such variations and consider the results on a relative basis. The crystallite size (*t*, Å) was calculated using the Scherrer eq (eq 2)^24^
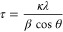
2where *k* is the Scherrer constant (0.94), and *l* is the wavelength of the X-ray radiation (1.54059). The full width at half-maximum (fwhm) of the diffraction peak is represented by *b*, in radians. *q* is the diffraction angle of the crystalline peak (020) in cellulose II and (200) in cellulose I.^22^ HFs’ orientation parameter (*f*) was calculated from the integration of the azimuthal intensities according to eq 3(17)
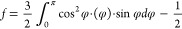
3where φ is the azimuthal angle (0–π) and *t(φ)* represents the normalized azimuthal intensity diffraction (eq S5) after subtracting the isotropic contribution.^17^

### Thermal Stability and Moisture Sorption

The thermal stability of HF was determined by thermogravimetric analysis (Thermo Gravimetric Analyzer Q500, TA Instruments) using a nitrogen atmosphere. The given HF was cut into small pieces before loading them in the TGA pan, and the samples were subsequently heated from 30 to 700 °C (10 °C/min heating ramp). The changes in mass as a function of temperature were recorded.

The moisture sorption abilities of MCC and HF were studied by using a dynamic vapor sorption device (surface measurement systems, DVS Intrinsic, UK) with an accuracy of 0.1 mg. MCC powder (21 mg) of HF samples (19 mg) was placed on the microbalance in the DVS chamber equipped with a relative humidity (RH) control. MCC and HF were preconditioned at 0% RH until they reached the equilibrium state. Moisture sorption was determined by recording the changes in mass as a function of RH at 25 °C and in a nitrogen environment. The adsorption and desorption behaviors were studied by RH cycling, from 0 to 5%, followed by a 10% increment, from 5 to 95%, and then back to 0%.

## Results and Discussion

### Cellulose Alignment and Rheological Behavior

**Figure 1:**
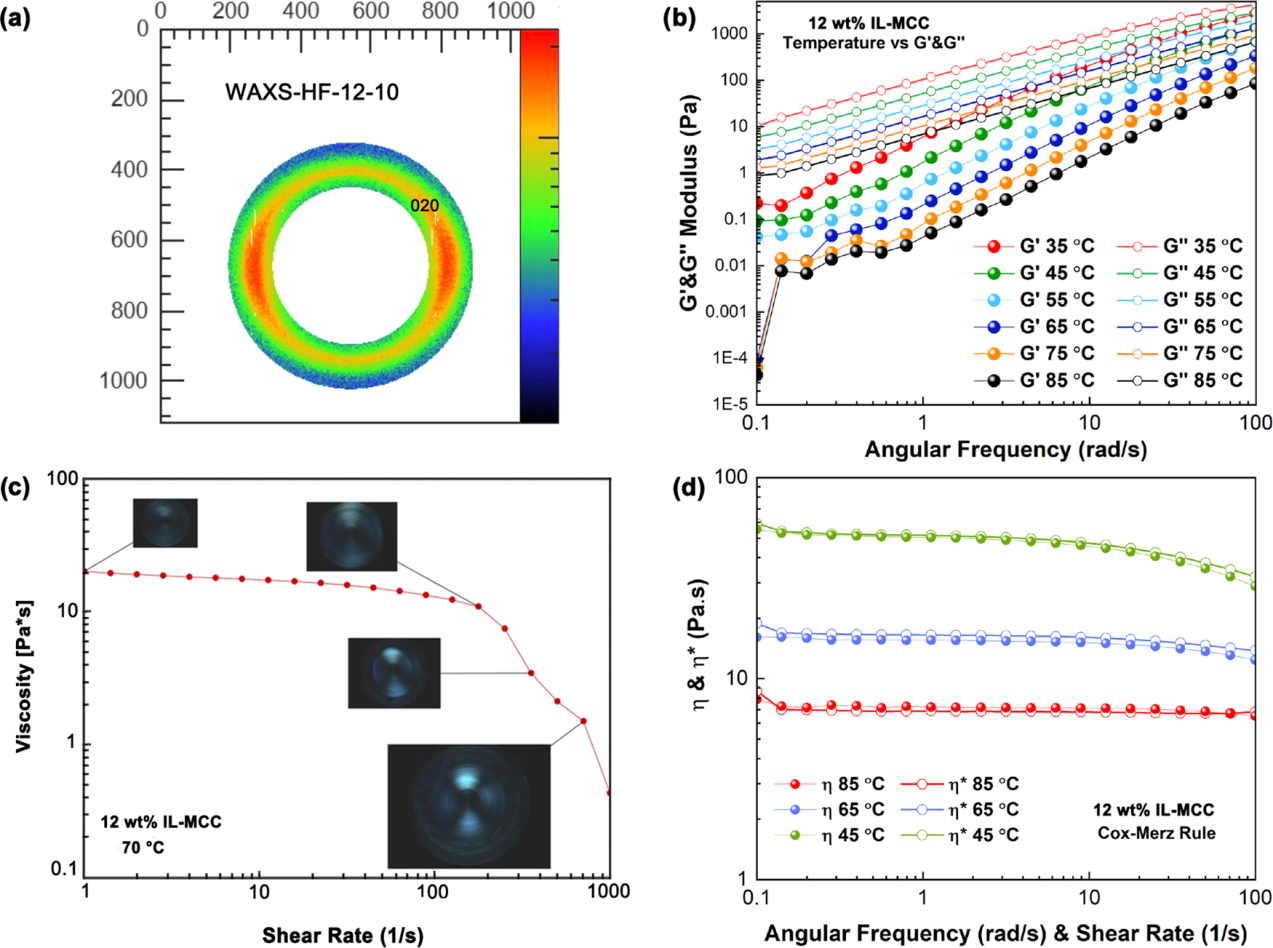
Flow properties of 12 wt % IL-MCC solutions and fiber alignment: (a) azimuthal intensities for HF-12–10. (b) Dynamic moduli from 35 to 85 °C with an increase of 10 °C. (c) Optical appearance under cross-polarized light, evidence of birefringence of IL-MCC solution under increased shear rate. (d) Cox–Merz rule at 45, 65, and 85 °C.

The orientation factor relates to the degree of alignment in the uniaxial direction of cellulose polymer chains within the filament; see Table 1 (note: a factor of one denotes full alignment, whereas a nil value implies the absence of orientation).^25^Figure 1a illustrates the integrated azimuthal main peak intensity of HF-12–10 and the corresponding fiber orientation factors (*f*) for samples HF-8–6 and HF-12–10, measuring 0.31 and 0.36 (Table 1). The results indicate a low degree of fiber orientation, which is associated with the viscous state of the dope, as observed in Figure 1b, and the absence of drawing during spinning^26^ (further explanation in the SI after Figure S5). Furthermore, the extrusion velocity (Table S1) of the cellulose solution had a negligible impact on fiber alignment, as evidenced by the birefringence intensity measured *in situ* (Figure 1c) and the Newtonian behavior (Figure 1d). In this context, the slightly improved orientation factor of HF-12–10 is conceivably induced by the increased air gap due to elongational stress and gravity^25^ and this outcome contributes to the extended mechanical strength of HF-12–10 and HF-12–12 (see the Thermal Stability and Strength section and Hauru et al.).^27^

**Table 1:** Structural Features of MCC and Hollow Filaments

sample	fwhm (deg)^a^	crystallite size (Å)	CrI (%)	*f*	air gap (cm)
MCC	1.7	49.9	82	-	-
**HF-**8–6	1.86	45.5	71	0.31	2
**HF-**12–10	2.3	36.9	64	0.36	3

a fwhm: full width at half-maximum.^24^

The crystallinity of HF (71 and 64%), Table 1, is lower than that of MCC (82%), noting that a high cellulose concentration results in a lower crystallinity index, with an evident transition to amorphous structures. This latter observation is a consequence of the transient cellulose reconstitution, where cellulose at high concentration may necessitate more time and energy to become ordered.^21^ Although by applying drawing or increasing air gap would promote a better alignment and mechanical strength at high cellulose concentrations,^28^ the increased amorphous contribution in HF improves toughness, as discussed in the following sections.

### HF Morphology and Molecular Structure

**Figure 2:**
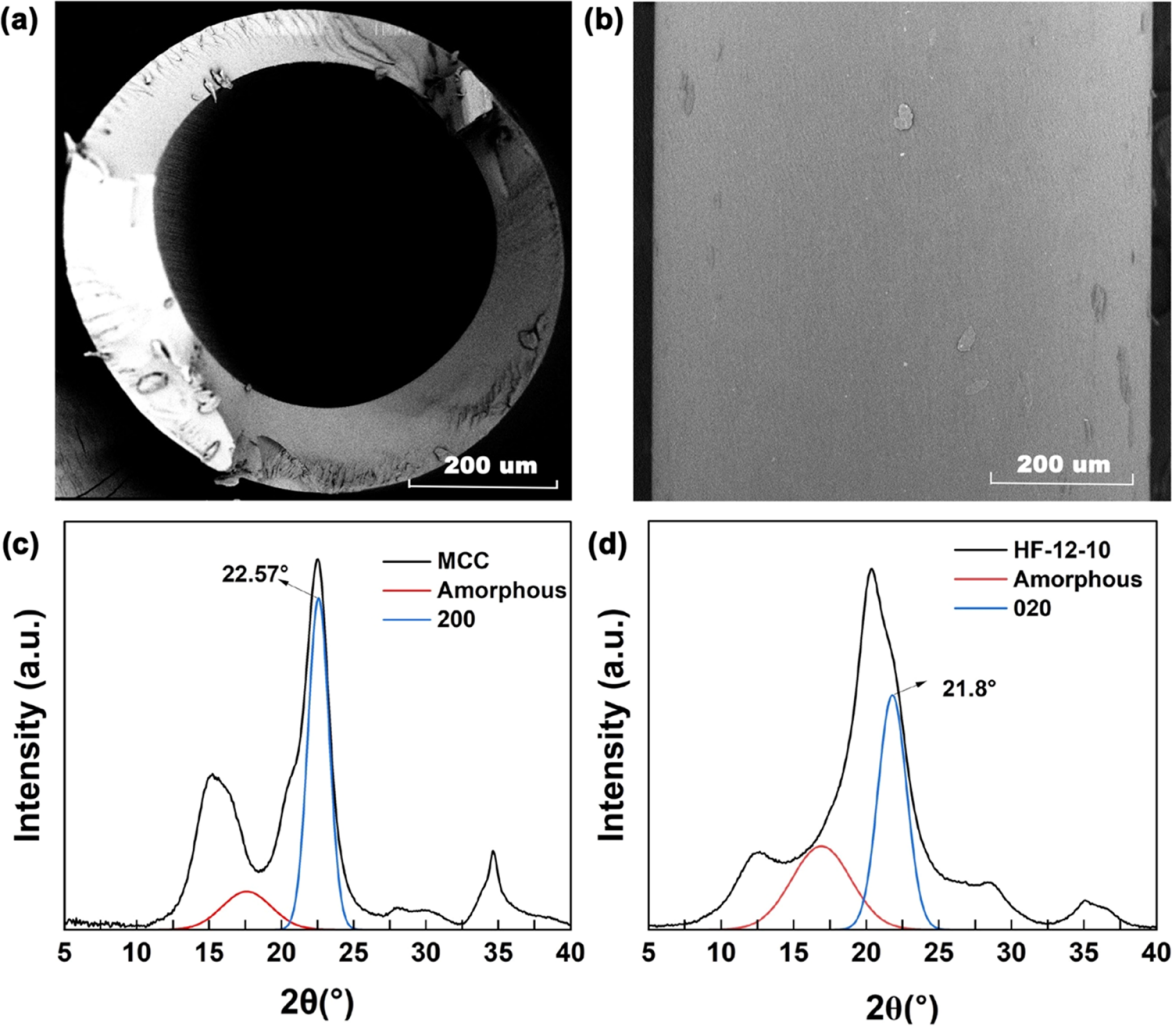
HF morphology, molecular, and crystal structure. (a) SEM image of HF cross-sectional morphology. (b) SEM image of HF surface topography. (c) MCC WAXS pattern. (d) HF WAXS pattern.

Well-defined shapes with a hollow core and dense wall were revealed in cross-sectional imaging of the HF (Figure 2a). The outer surfaces of the filaments were smooth (Figure 2b) and presented a uniform topography (Figure S6), evidencing that the nascent HF stabilizes with the instant solvent exchange, promoted upon contact with the coagulant. No defects were apparent, Figure 2b, in agreement with the study by Falca et al.^29^ who revealed that phase inversion of an IL cellulose solution in water produces a dense and smooth surface morphology. This superior HF morphology, as compared with the existing HF systems,^30,31^ confirms the benefits of water regeneration from the [emim][OAc] solution.

The crystal structure revealed by WAXS showed the typical crystal plane diffraction patterns of cellulose I (MCC) and cellulose II (HF), Figure 2c,d, respectively.^22,26^ The alteration of crystalline diffractions coincides with the FTIR results (Figure S7), implying that the regenerated cellulose underwent only a structural transformation from cellulose I to cellulose II, without derivatization. The peak at 1640.94 cm^–1^ (Figure S7) assigned to the O–H band in the amorphous region only appeared in HF but not in MCC, suggesting water molecules exist in the disordered areas of the cellulose II backbone.^28^ In this context, the impact of water molecules’ interaction with the cellulose structural transition affects the material characteristics, as discussed in terms of HF’s mechanical performance and dynamic water sorption behavior. Additionally, the superior solubility, noncorrosiveness, and exceptional handling safety of the IL [emim][OAc] offer greater value compared to traditional solvent systems for HF fabrication and provide HF with toughness, reaching or exceeding current achieved levels.^19,32,33^

### Thermal Stability and Strength

The TGA isotherms of MCC and HF were measured (Figure S8) and the respective degradation temperatures (Table S2) suggest that the HF have a relatively lower thermal stability compared to MCC; this difference is attributed to alterations in the crystal structure following dissolution in the IL and subsequent water regeneration, leading to a decrease in crystallinity (Table 1).

**Figure 3:**
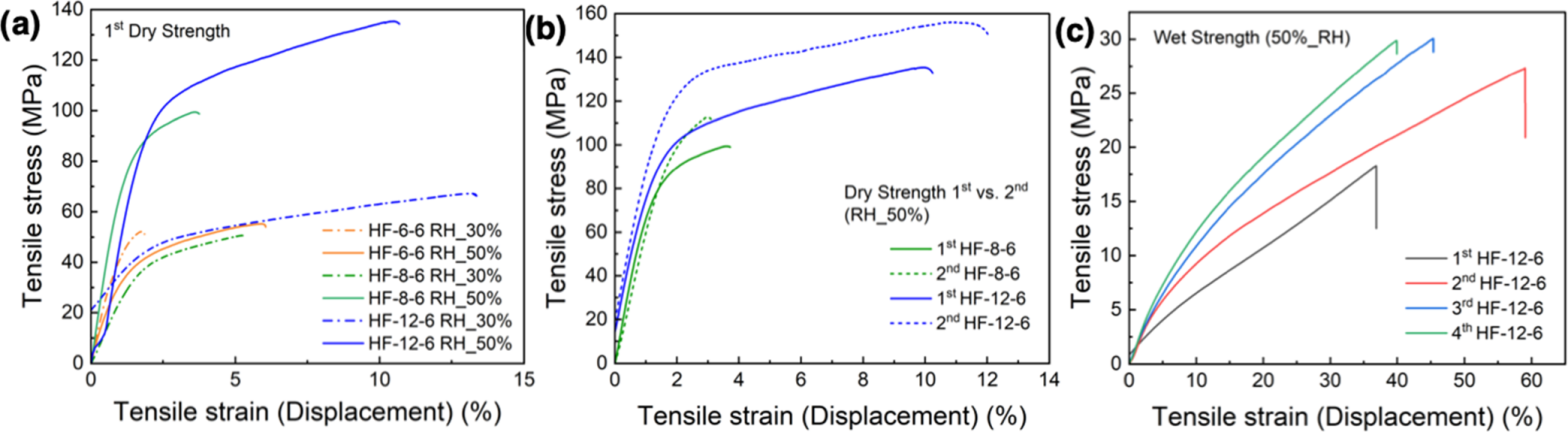
Mechanical performance of hollow filaments: (a) HF mechanical performance after drying at 30 and 50% RH. (b) HF dry mechanical performance after cyclic wetting. (c) HF mechanical performance in the wet states after cyclic wetting.

Figure 3 depicts the mechanical performance of HF in both dry and wet conditions; it becomes evident that filament stretchability increases with cellulose concentration in the dope (Figures 3a,b and S9a,b). Samples HF-6–6 and HF-8–6 showed much less strain (Figure 3a), which is associated with their high crystallinity index (a higher crystal content leads to higher stiffness and brittleness).^25^ HF-6–6, HF-8–6, and HF-12–6 tensile test profiles were compared after drying under room conditions (23 °C, 30% RH) and in a conditioned environment (23 °C, 50% RH), as shown in Figure 3a. The results indicate that drying at a slower rate in higher humidity environments promotes the formation of stronger interfibrillar bonds, positively influencing the mechanical performance.^34^ The results also confirm that a higher cellulose concentration in the dope results in denser and thicker HF wall layers, with improved mechanical performance.^35^ As expected, the wet strength was lower than the dry value. For instance, the wet strength of HF-12–6 dropped to 18.2 MPa (13% reduction), following a wetting and drying cycle. Nevertheless, the wet hollow filaments exhibited outstanding elongation (Figure S9a,b): HF-12–6 reaches up to 40% strain (Figure S9a) and the strain of HF with the lowest cellulose concentration (HF-6–6) surpasses that of all dry samples (Figures S9a and 3a,b)

Overall, both an increased tensile strength and strain were observed after cyclic wetting and drying (Figures 3b,c and S9b–d). Sample HF-12–6 reached the highest modulus (160 MPa), wet strength (30 MPa), and strain (60%). In addition, there was no significant reduction in the wet strength after four drying and wetting cycles. This implies that the interaction between water and regenerated cellulose plays a prominent role, potentially due to the molecular diffusion and interfibrillar lubrication, resulting in the HF’s flexibility. This latter observation agrees with the FTIR results that evidenced the presence of water molecules in the amorphous region of HF.

An outstanding stretchability was noted for HF (HF-12–10 and HF-12–12 using a 3 cm air gap, Figure S9c,d), reaching a value of up to 80% in the wet state (40% in the dry state). However, a decrease in the dry and wet tensile strength occurred as the extrusion velocities were beyond 6 mL/min. Previously, it was stated that the spinning rate of the cellulose solution did not exhibit a direct impact on fiber alignment (Figure 1b–d and Table S1); therefore, HF tenacity should be independent of the solution extrusion rate at a constant drawing.^27^ However, our results suggest that the physical properties of HF were affected by the pumping rate of the bore fluid (*viz*., deionized water), producing defective layers, with poor mechanical performance,^21^ as revealed in the tensile tests (Figure S9c,d). In addition, higher extrusion rates increase the inner HF diameter and reduce the wall thickness,^36,37^ contributing to HF tenacity reduction (HF-12–10 and HF-12–12 samples, Figure S9c,d). Nevertheless, the tensile strength, moduli, and tenacity (Tables S3 and S4) surpass the values reported so far;^29,38,39^ see Table S5. The excellent physical–mechanical properties of HF in both dry and wet states are believed to result from the predominant role of water interactions between the cellulose structure and IL, further investigated through measurements relevant to the interactions with water, discussed next.

### Water Sorption Behavior

**Figure 4:**
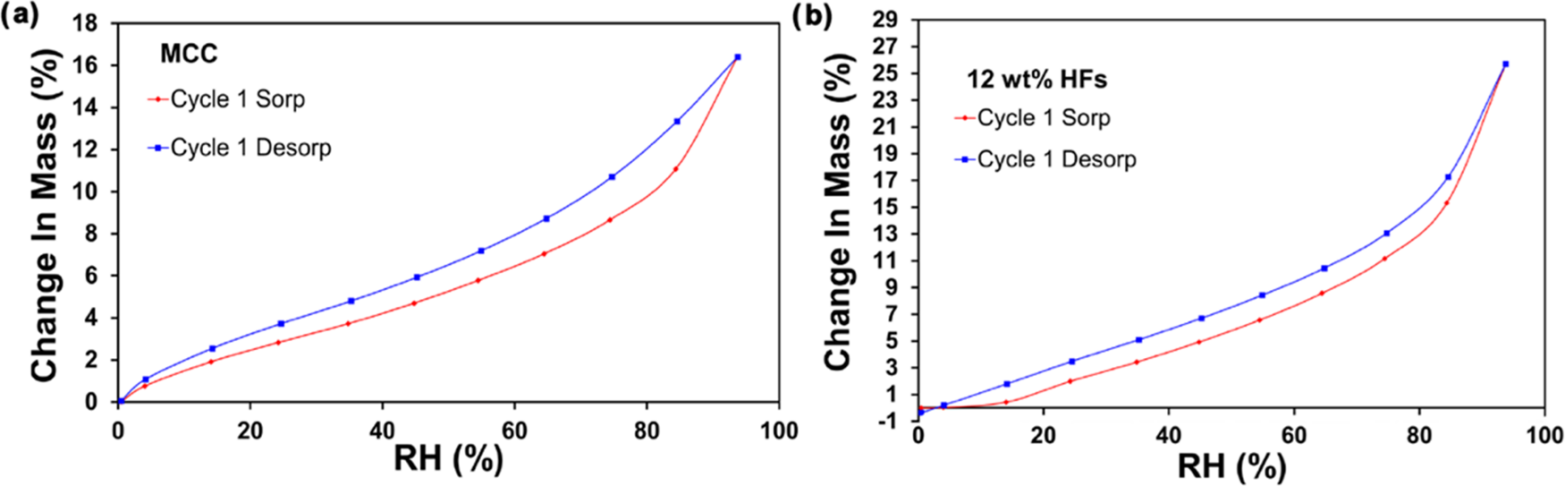
Moisture sorption ability of MCC and HF: (a) Adsorption and desorption isotherms of MCC. (b) Adsorption and desorption isotherms of HF-12–6.

The tensile strength and strain of hollow filaments in the dry and wet states are enhanced upon being subjected to drying and wetting cycles. Here, water vapor sorption (WVS) and desorption (WVD) measurements can help to understand the underlying mechanisms. The moisture adsorption and desorption isotherms of MCC and HF are shown as a function of the equilibrium moisture content (EMC) (Figure 4a,b; see Table S6 for statistics). The EMC of HF was 25.7% at 95% RH. In contrast, the corresponding value for MCC was 16.4%, indicating that the HF exhibits a larger number of available hydrophilic sites,^40^ which is attributed to the increased cellulose II content and decreased crystallinity.^41^ On the contrary, higher crystallinity leads to strong interfibrillar bond networks, limiting water sorption. In this context, water molecules adsorb mainly on the surface for highly crystallized cellulose samples; however, they can penetrate in the amorphous regions.^41,42^

MCC samples exhibited typical capillary condensation after RH reached 80%.^41,42^ Hatakeyama and Hatakeyama^43^ revealed that the amorphous units in cellulose I tend to be more organized in the presence of small amounts of water; in contrast, the amorphous units in cellulose II become less organized, and the amorphous portion expands gradually as moisture increases, explaining why the filaments have better WVS ability and swell to a greater extent as compared to MCC. Moreover, water vapor penetration into cellulose matrices lowers the stiffness of inter- and intramolecular networks,^41,42^ increasing stretchability. MCC and HF EMC isotherms (Figure S10a and b) demonstrate that the morphology and structure of the samples also influenced the total adsorption time, until reaching the EMC.^44^ In summary, cellulose polymorph transition and the increased amorphous regions enabled interfibrillar lubrication, positively impacting the HF tensile strength and resiliency. Therefore, the strength and elongation of HF in the dry and wet states were further extended with the addition of drying and wetting cycles. In related efforts, Cieśla et al. (2004) indicated three key water interactions occurring in the presence of regenerated cellulose: these are bulk water interaction (or “free” water), interfacial water interaction (freezable bound water), and strongly bound water (or so-called nonfreezable water).^45^ Significantly, the observation area is in agreement with the TGA results (Figure S8) that revealed that small peaks under 200 °C are attributable to the HF interfacial water and strongly bound water. In addition, nonfreezable water connected to the cellulose backbone (due to the presence of the three hydrophilic hydroxyl groups on the equatorial direction of a glucopyranose ring of cellulose)^46^ increases as the regenerated cellulose spends more time in water, positively impacting the sorption ability of HF at high RH (Figure 4b). Consequently, increased tenacity of HF in the conditioned treatments is achieved (drying under RH 50% and rewetting circles). Moreover, the changes in the swelling of HF may lead to crystalline ordering in the cellulose fractions as a result of the greater binding strength between water and HF.^45^ The hydrophilic characteristics and water interactions of HF contribute positively to the mechanical performance.

## Conclusions

A platform to produce hollow filaments (HF) from cellulose regenerated from ionic liquid dissolutions was introduced by using coaxial dry–jet wet spinning. The cellulose HF exhibited remarkably high wet strength (30 MPa) and stretchability (80% strain), which makes it suitable for applications requiring wet performance. The impressive physical and mechanical characteristics of HF in its wet state arise from the interactions between water and the regenerated cellulose, leading to structural reordering. The developed HFs are relevant to applications encompassing the areas of life sciences (stem cells, nerve reparation), capillaries, vessels, and tubing for separation technologies (permeation membranes, and others). Further efforts should focus on the variation of HF pore size distribution, aiming at applications such as water purification and filtration. Moreover, *in vivo* and *in vitro* cell culture studies might be considered for utilizations in bioreactors or in functional scaffold for the production of extracellular vesicles.

## Supporting Information Available

Fabrication method and cellulose solution of HF; viscoelasticity of cellulose solution; HF physical appearance; FTIR and TGA results; HF mechanical properties; and dynamic vapor sorption results (PDF)
