# Supplementary material for: Hollow Filaments from Coaxial Dry–Jet Wet Spinning of a Cellulose Solution in an Ionic Liquid: Wet-Strength and Water Interactions
Source: Biomacromolecules. 2023 Dec 12;25(1):282–9. doi: 10.1021/acs.biomac.3c00984 (PMC10777343; doi:10.1021/acs.biomac.3c00984)
Supplement: Supplementary file 1 — bm3c00984_si_001.pdf [file bm3c00984_si_001.pdf]

Supporting Information

Hollow Filaments from Coaxial Dry-Jet Wet Spinning of a Cellulose Solution in an Ionic Liquid: Wet-Strength and Water Interactions

Shiying Zhang[1]*, Guillermo Reyes*[1*]*, Alexey Khakalo*[2]*, Orlando J. Rojas*[3*]

[1] Department of Bioproducts and Biosystems, School of Chemical Engineering, Aalto University, FI-02150, Espoo, Finland

[2] VTT Technical Research Center of Finland, FI-02150, Espoo, Finland

[3] Bioproducts Institute, Department of Chemical & Biological Engineering, Department of Chemistry and Department of Wood Science, The University of British Columbia, 2360 East Mall, Vancouver, BC V6T 1Z3, Canada

*Corresponding author: Orlando J. Rojas, Email: orlando.rojas@ubc.ca

Guillermo Reyes, Email: guillermo.reyes@aalto.fi

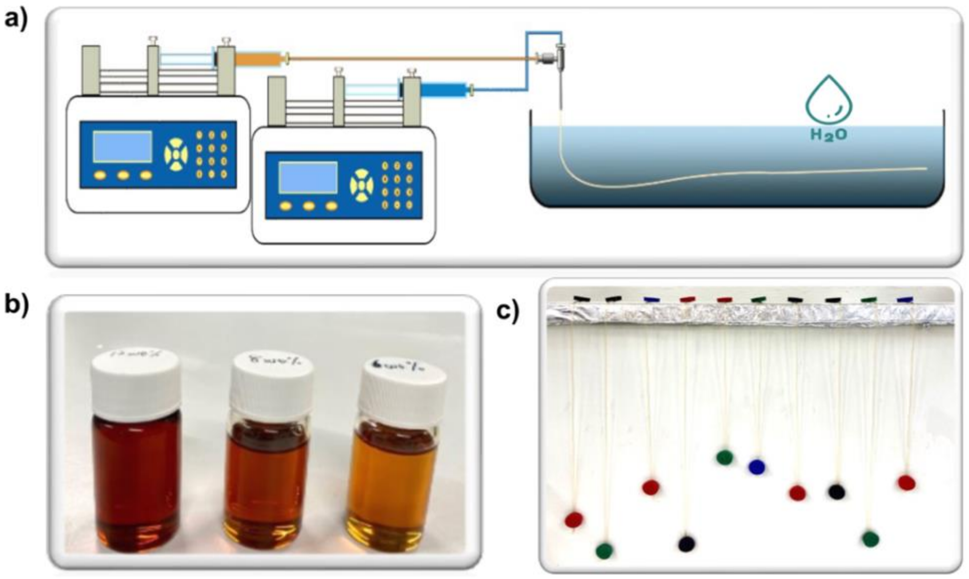

Figure S1. HF spinning and drying. a) coaxial dry jet-wet spinning set-up for HF production. b) [emim][OAc]-MCC solutions at 12, 8 and 6 wt% cellulose percentage from left to right respectively. c.) HF drying under tension.

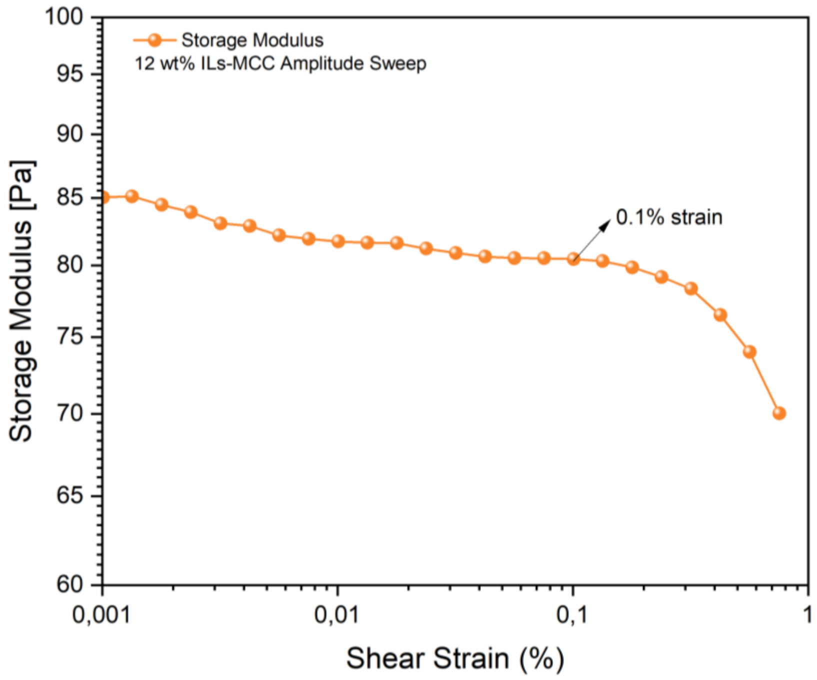

Figure S2. Amplitude-sweep of 12 wt% cellulose solution measured at 50 °C, 0.1 % strain was determined by plotting storage modulus as a function of shear strain within the linear range.

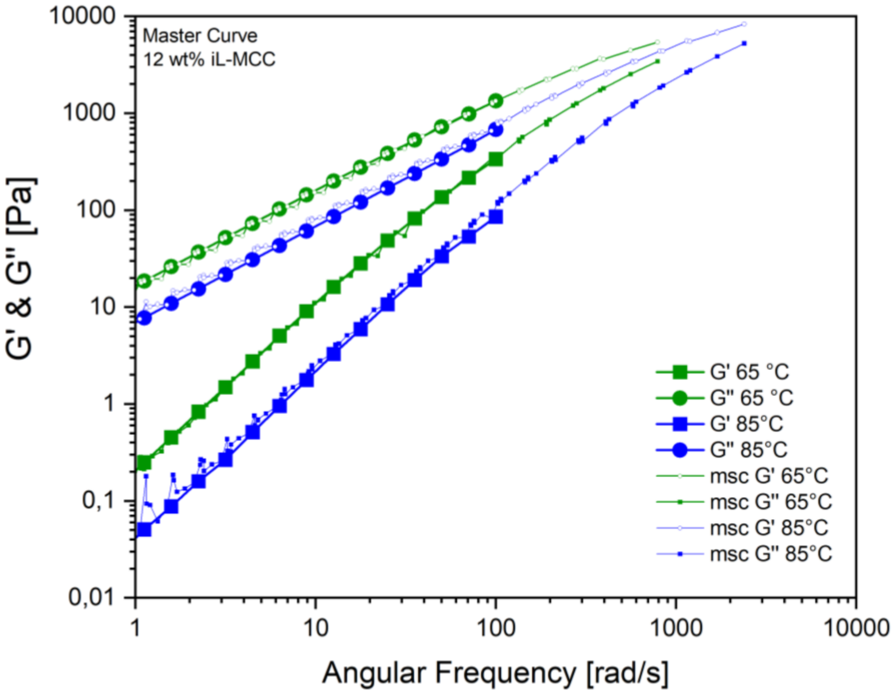

Figure S3. Master curve of dynamic moduli of 12 wt% [emim][OAc]-MCC solution at 65°C and 85°C in the high angular frequency range.

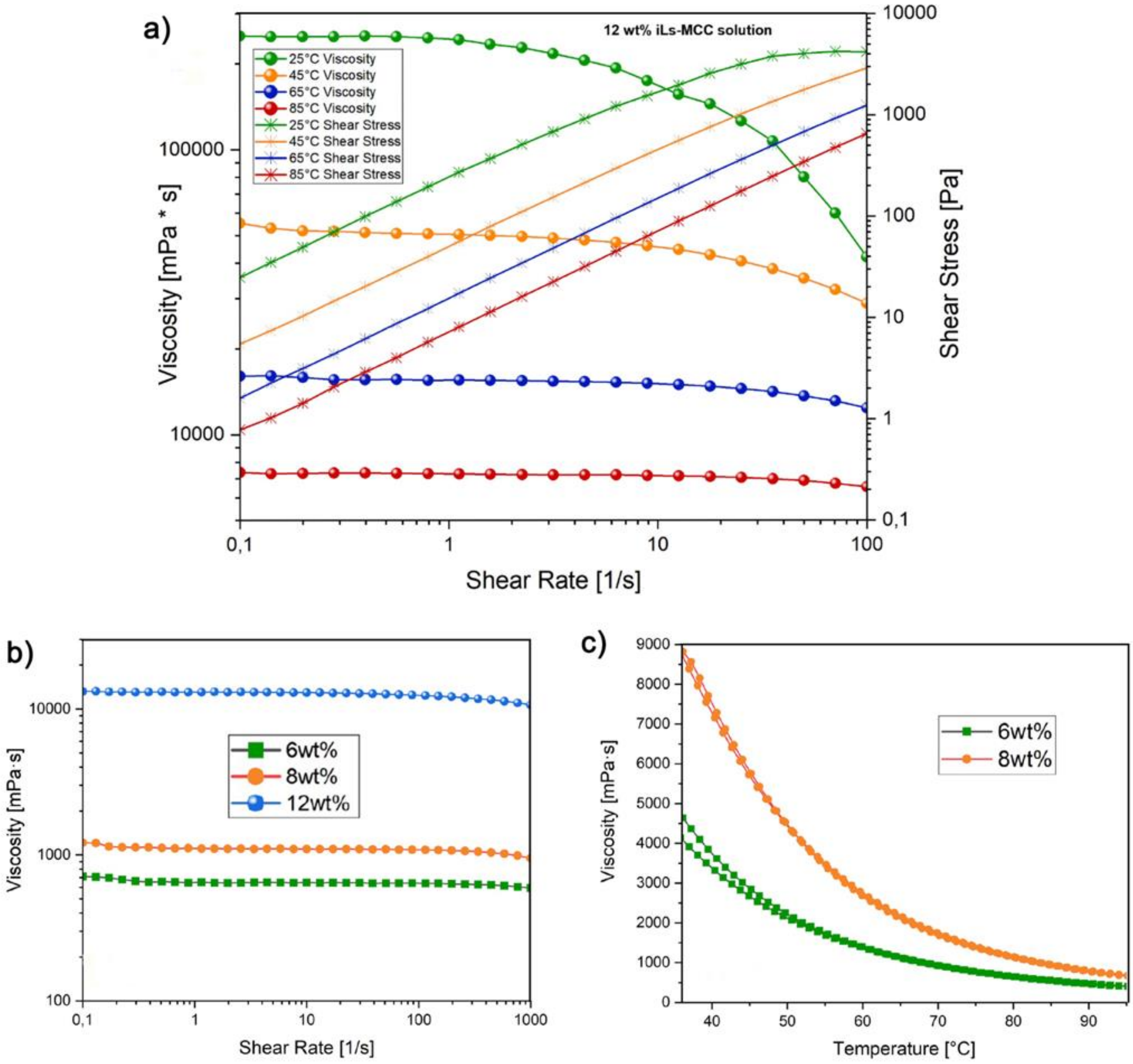

Figure S4. a) viscosity and shear stress as a function of shear rate measured at 25 °C, 45 °C, 65 °C, and 85 °C of 12 wt% cellulose solution. b) viscosity of 6 wt%, 8 wt%, and 12 wt% dope as a function of shear rate measured at 80 °C. c) the viscosity of 6 wt% and 8 wt% IL-MCC solutions as a function of temperature from 35 to 95 °C then back to 35 °C measured at a constant shear rate of 100 s^{-1}.

The viscoelastic behavior of the polymer solutions determines their spinnability and the corresponding physical-mechanical properties after regeneration. According to the results of shear viscosity **(Figure S4)** and the dynamic moduli (**Figure 1b),** the viscosity of the cellulose solution is dependent on the cellulose concentration and temperature. The viscous behavior is

predominant since the storage modulus (G') did not surpass the loss modulus (G") during the whole measuring range.[1] However, as the angular frequency increases, G' approaches G", indicating the disentanglement tendency of cellulose polymer chains to a fixed elastic network when G' exceeds G".[1] The master curve of dynamic moduli (**Figure S3**) further predicted the visco-elastic behavior of the cellulose solution at a high angular frequency. The viscosity decreased significantly as the temperature increased (**Figure S4**), and the long-range Newtonian behavior (**Figure S4a-b,** and **1c-d**) implies that the shear rate has less influence on cellulose chain deformation. It is conceivable that the air gap induced the fiber orientation factor of the HF (**Table 1**) due to elongational stress and gravity.[2] The shear viscosity ($\eta|\dot{\gamma}$) flow curves closely agree with the complex viscosity ($\eta^*|\omega$) at 45 °C to 85 °C (**Figure 1d**), indicating that the empirical Cox-Merz rule was satisfied.[3]

Table S1. The Shear Rate of the Corresponding Solution Extrusion Velocity

Extrusion velocity (mL/min)	Shear Rate (1/s)
**6**	291
**10**	482
**12**	578

*All the shear rate of the cellulose solution pump rate (80 °C) were within the Newtonian range.

As a result of the findings in **Table S1**, one can argue that the extrusion shear rate has a limited influence on fiber alignment. Because the cellulose solution exhibits little deformation at extrusion speeds (6-12 mL/min).

The respective shear rate of the extrusion velocity is calculated according to **Equation S1**[4]**,** the shear rate depended on the inner diameter of the coaxial needles.

$$\gamma = \frac{4Q}{\pi R^3} \quad \text{(S1)}$$

Where γ is the shear rate, Q is the volumetric rate (solution extrusion velocity), and R is the inner radius of the spinning needle.

The equivalent HF and spinneret diameter were calculated by **Equation S2** since the HF was fabricated by the coaxial spinning needles.

$D_e = (D^2_{outer} - D^2_{inner})^{1/2}$ (S2)

Where D_{outer} is the diameter of the larger spinning needle and D_{inner} is the smaller spinning needle.

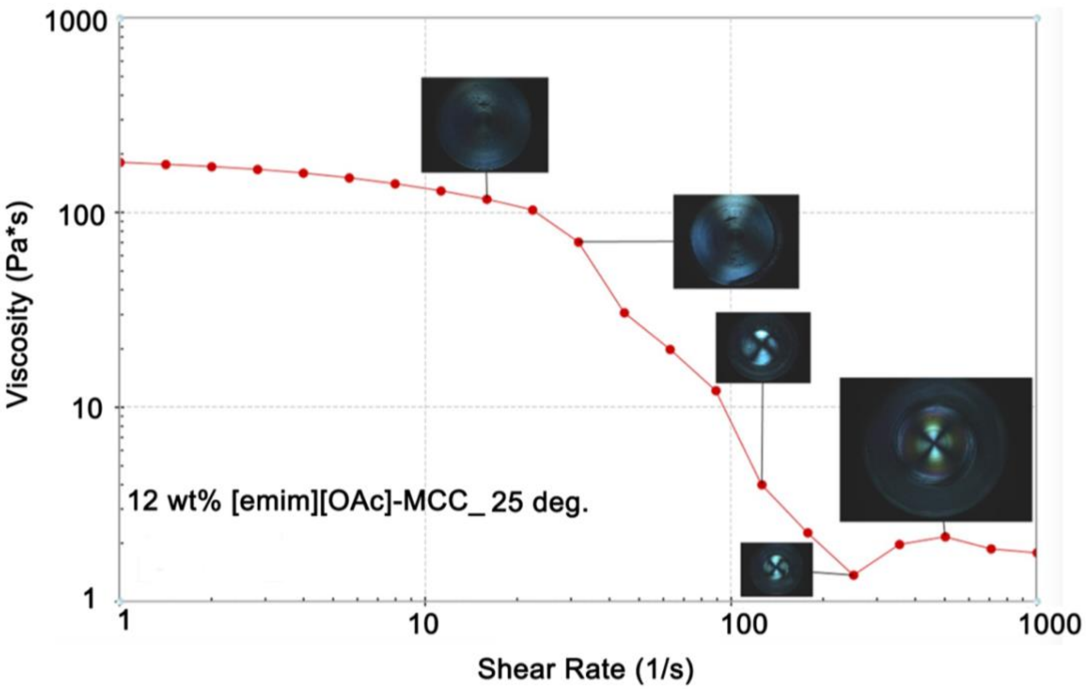

Figure S5. Shear viscosity measurement with polarizing microscopy of 12 wt% MCC solutions at 25 °C within the shear rate range of 1 s^{-1} to 1000 s^{-1}.

Compared to **Figure 1c, Figure S5** clearly shows that the [emim][OAc]-MCC solution at a low temperature (25 °C) exhibits a high degree of shear thinning at a high shear rate (> 10/s). The significant birefringence in **Figure S5** further confirms that [emim][OAc]-MCC solution at high temperature has less deformation due to the low viscosity. Particularly, the [emim][OAc]-MCC solution shows a second Newtonian behavior after the shear rate is higher than 200/s.

The birefringence was used as a practical and *in-situ* method to observe the deformation and alignment of polymer chains within the polymer solution under shear rate. During the extrusion

of the cellulose solution through the coaxial spinning needle, the system was subjected to shear (the latter can be calculated from the extrusion velocity, as outlined in Table S1). The birefringence demonstrates that during the extrusion (spinning) process, the polymer solution remained in a viscous state. This implies that no deformation or alignment of the cellulose polymer chains occurred. This serves as evidence supporting the statement that the extrusion rate of the solution had no significant effect on the fiber alignment (orientation factor) of the HF.

Typically, polymer solutions undergo a transition from a viscous to an elastic state under an increased shear rate. This transition means a more aligned state at increased shear rate. However, in our case, the cellulose solution remained in a viscous state at the applied extrusion velocity.

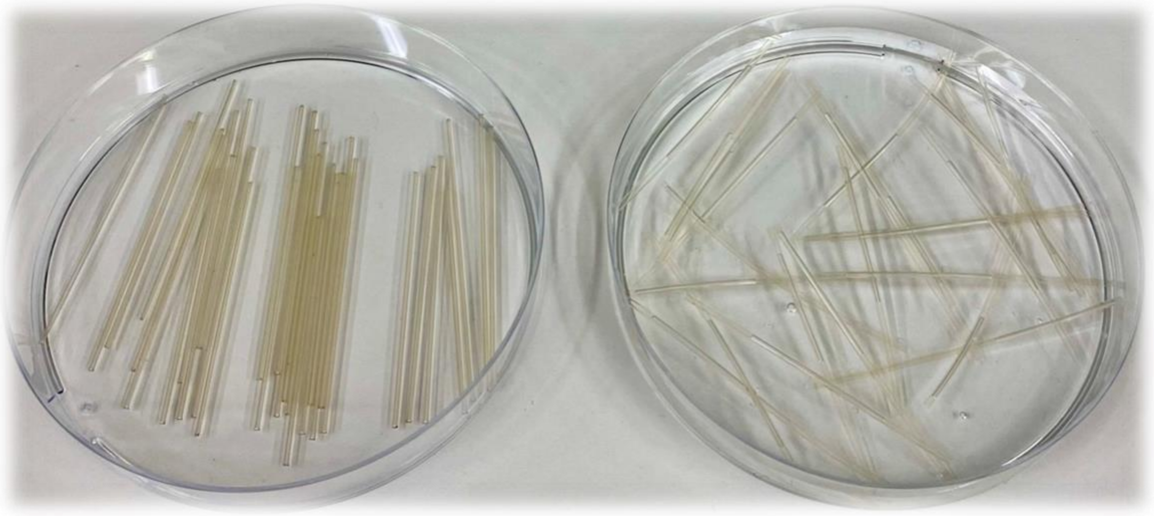

Figure S6. HF physical appearance in dry (left one) and wet (right one) state.

The tensile strength of the HF was determined according to the **Equation S3**.

$$\sigma = \frac{\mathrm{F}}{\pi * (R1^2 - R2^2)} \quad \text{(S3)}$$

Where σ is the yield tensile strength, F is the force that applied to the HF before its plastic deformation, and R_1 and R_2 are the radius of the HF and the core, respectively (see the illustration below, **Figure S6s**).

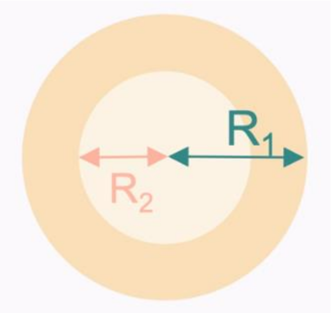

Figure S6s. The illustration of the cross-section radius of the HF, the true area under force is in yellow.

The elastic modulus of the HF was calculated according to the **Equation S4**.

$$E = \frac{\sigma}{\varepsilon} \quad \text{(S4)}$$

Where E is the Young's modulus, σ is the stress, and ε is the strain.

The reflection peak being evaluated is the main peak (020).

The normalized azimuthal distribution $\tau(\varphi)$ is calculated as the **Equation S5**.

$$\tau(\varphi) = \frac{I\ (\varphi)}{\int_0^{\pi} cos^2\varphi \cdot \tau(\varphi) \cdot sin\varphi d\varphi - \frac{1}{2}} \quad \text{(S5)}$$

Where I is the intensity of diffraction peaks from 0- π (φ).

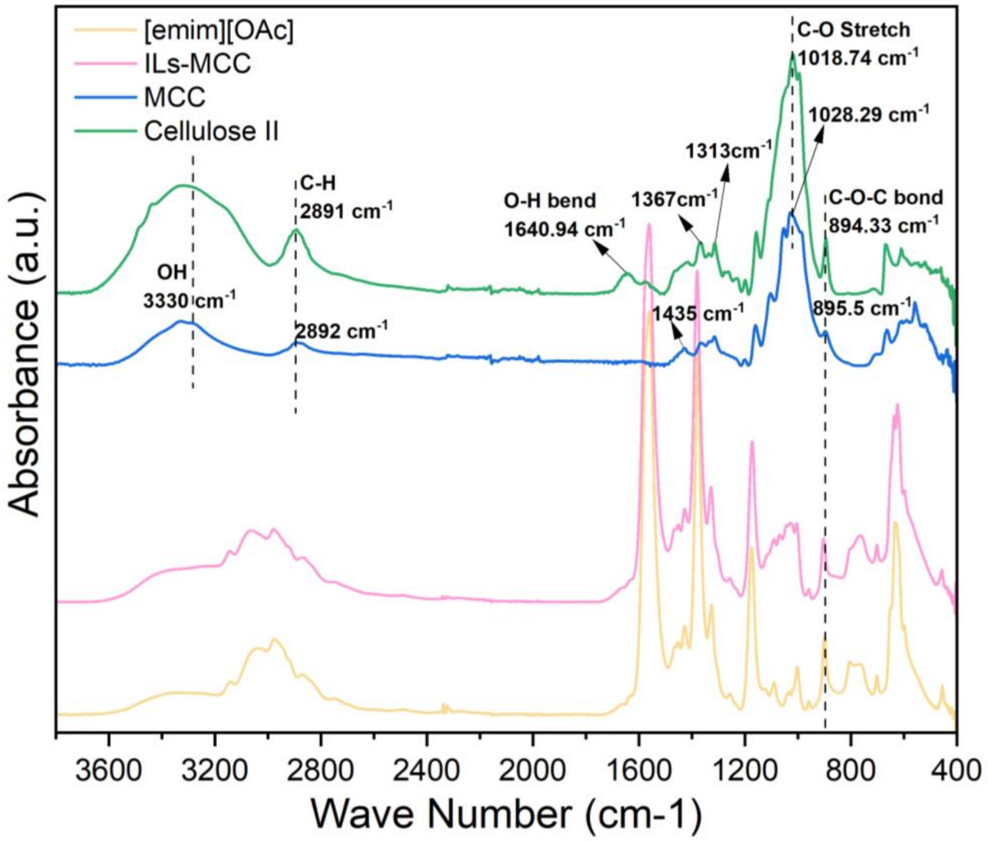

Figure S7. FTIR-ATR spectra for MCC, HF (cellulose II), ILs-MCC solution, and IL [emim][OAc].

The FTIR spectra (**Figure S7**) reveal a high similarity between the MCC and regenerated HFs samples, implying that only physical changes and polymorph transitions occurred during the cellulose dissolution and regeneration in [emim][OAc] solvent.[5] The peak at 894.33 cm^{-1} in the cellulose II spectrum representing C-O-C stretching vibration in the amorphous regime became more intense than the MCC, meaning that glycosidic linkage destruction occurred during cellulose dissolution, [6-7] and the regime of dislocated cellulose chains increased. Moreover, the skeletal vibrations from the C-O stretching band appeared at 1028.29 cm^{-1} in MCC with a small shoulder on the left. It did, however, slightly shift to 1018.74 cm^{-1} in cellulose II without the small shoulder, demonstrating that the connection in the hydrogen-bonding system varied when cellulose I was transformed to cellulose II. [8] Furthermore, the crystal transition to cellulose II can be noticed from the peaks located at 1313 cm^{-1}, 1367.3 cm^{-}

[1], and 1453 cm^{-1}, correspondingly elucidated as O-H, C-H in-plane bending increased and the CH_2 scissoring vibration at C6 weakened when compared to cellulose I. [7-8]

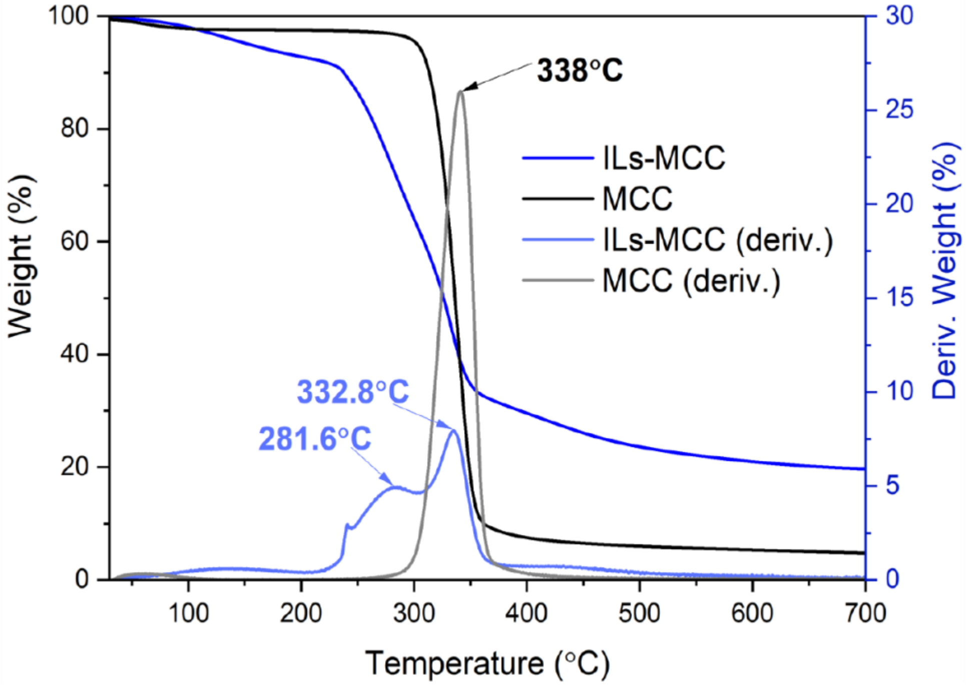

Figure S8. MCC and HF's thermal stability.

The curves of weight loss and derivative weight loss as a function of temperature in **Figure S8** show the thermal behaviors of HF compared to MCC powder. The MCC and HF isotherms revealed a slight weight loss below 100 °C due to the cellulose structure's dehydration of free water molecules. [5] According to the results (summarized in **Table S2)**, the degradation of MCC initially occurred at 260 °C, then dramatically reached the maximum weight loss at 338 °C, which is consistent with the reported degradation of pure cellulose.[5] Whereas the onset degradation temperature of HF is 220 °C followed by a small degradation peak at 281.6 °C and the maximum weight loss at 332.8 °C, which coincides with the observation of Tan et al. that the pyranose rings in cellulose II decompose after the glycosidic linkages are open.

Table S2. Degradation Temperatures and Decomposition Peaks for MCC and HF Samples.

**Sample**	T_{onset} (°C)	T_{peak} (°C)
**MCC**	260	338
**HF**	220	281.6 and 332.8

Other minor temperature differences between MCC and HF in their TGA profiles are attributable to the increased amorphous cellulose composition in HF and the converted hydrogen bonding network. The results suggest that the HF retain relatively less thermal stability compared to MCC, which is related to the crystal structure ordering after dissolution in IL [emim][OAc] and water regeneration.

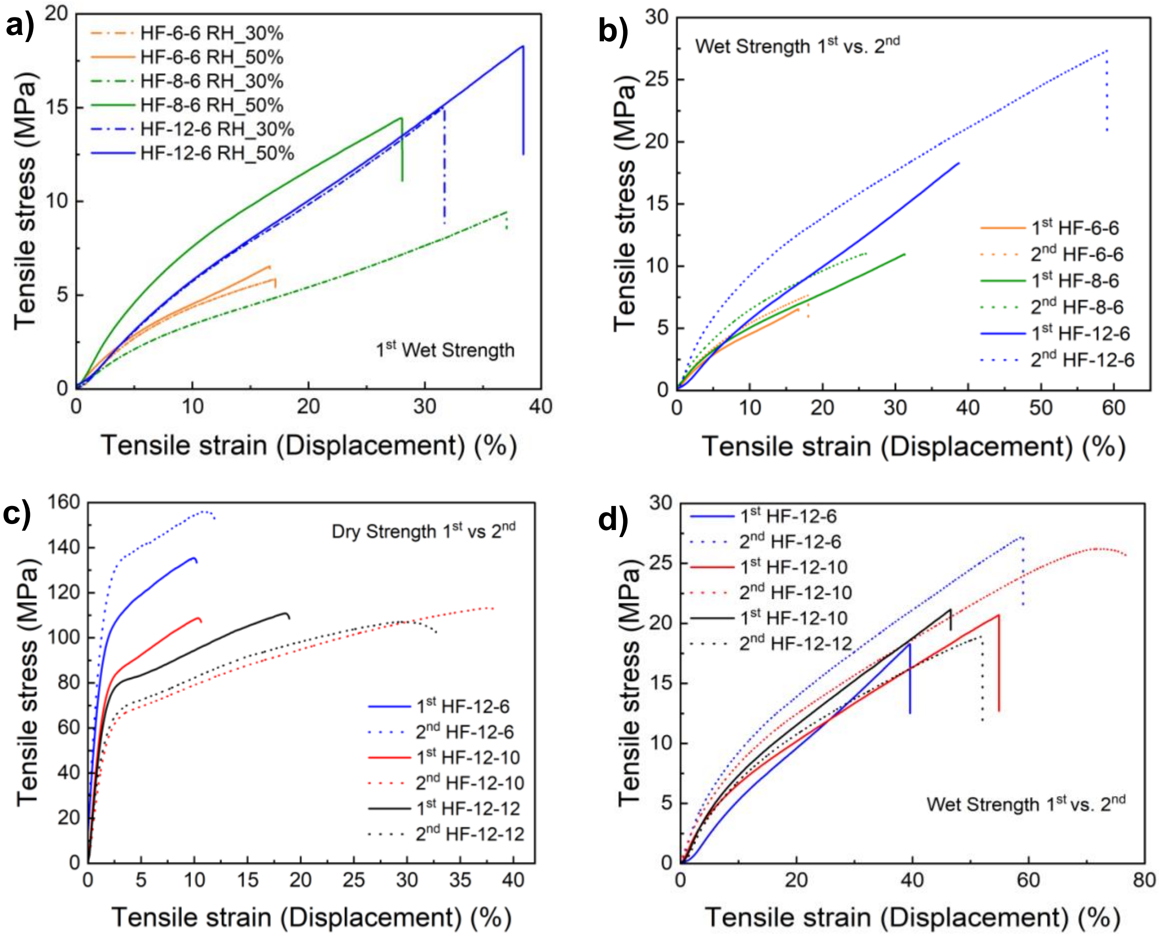

Figure S9. a) the initial wet strength of HF-6-6, HF-8-6, and HF-12-6 after drying in a normal room (RH_30%, T_23 °C) and a conditioned environment (RH_50%, T_23 °C). b) the initial

and repeated wet strength of HF-6-6, HF-8-6, and HF-12-6. c and d) the initial dry and wet strength and the (2nd) redrying and rewetting strength (under 50% RH_23 °C condition) of the HFs-12 spun at 6 mL/min, 10 mL/min, and 12 mL/min respectively.

Table S3. HF Mechanical Parameters

Sample	Modulus (GPa)	Strength (MPa)	Strain (%)	Toughness* (J)	Air Gap (cm)
**HF-6-6_dry**	4.8	58	6	264.1	2
**HF-6-6_wet**	0.072	6.53	17	84.32	2
**HF-8-6_dry**	6.37	112.58	3	283.5	2
**HF-8-6_wet**	0.109	14.43	27	182.5	2
**HF-12-6_dry**	7.38	156	12	1599.4	2
**HF-12-6_wet**	0.131	27.25	60	986.7	2

Toughness* is the area under the stress-train curve, calculated by integrating the stress-strain curve.

Table S4. Dry and Wet Strength of HF-12.

Sample	Modulus (GPa)	Strength (MPa)	Strain (%)	Toughness* (J)	Air Gap (cm)
**Dry_HF-12-6**	7.38	156	12	1599.4	2
**Wet_HF-12-6**	0.131	27.25	60	986.65	2
**Dry_HF-12-10**	3.896	113.2	39	3443.99	3
**Wet_HF-12-10**	0.102	26.2	80	1333.1	3
**Dry_HF-12-12**	4.71	110.2	33	2899.3	3
**Wet_HF-12-12**	0.1	18.9	52	612.36	3

Toughness* is the area under the stress-strain curve, calculated by integrating the stress-strain curve.

Table S5. The Spinning Systems and Properties of HFs.

Type	Material	Solvent	Method	Application	Tensile Strength (MPa)	Strain (%)	Ref.
Self-stand HF	Cellulose	[EMIM][OAc]	Dry-wet spinning	UF/NF dye separation	21	12	(9)
Self-stand HF	Cellulose	[EMIM][DEP]	Dry-wet spinning	UF/NF dye separation	30	74	(9)
Self-stand HF	Cellulose	[DMIM][DEP]	Dry-wet spinning	UF/NF dye separation	23	36	(9)
Self-stand HF	Cellulose	NMMO/PEG-400/n-propyl gallate	Dry-wet spinning	UF oil-water separation	-	-	(10)
Self-stand HF	PES	[EMIM][DEP]	Dry-wet spinning	DNA separation	4±0.3	51±12	(11)
Self-stand HF	PES	DMF	Dry-wet spinning	DNA separation	6±1.3	84±56	(11)
Self-stand HF	FEP	DMAc/PEG-400/PVP-K30/	Wet spinning	Textile waste treatment	18.5	11	(12)

Reinforced HF	PST/PET twisted fiber bundle	DMAc/PEG-400/PVP-K30/	Dry-wet spinning	Textile waste treatment	140.4-185.7 (Dry)	10-12	(12)
Self-stand HF	Cellulose Acetate	[EMIM][OAc]/ Acetone	Dry-wet spinning	Water permeance	0.25	-	(13)
Dual-layer HF	PES/PFI/PEI	NMP/DMF/DMAc	Co-extrusion	Gas separation	-	-	(14)
Hybrid braid reinforced HF	Cellulose acetate/PAN	DMAc/PEG-2000	Dry-wet spinning	TOC removal	31.5-33.8	40.4-47.2	(15)

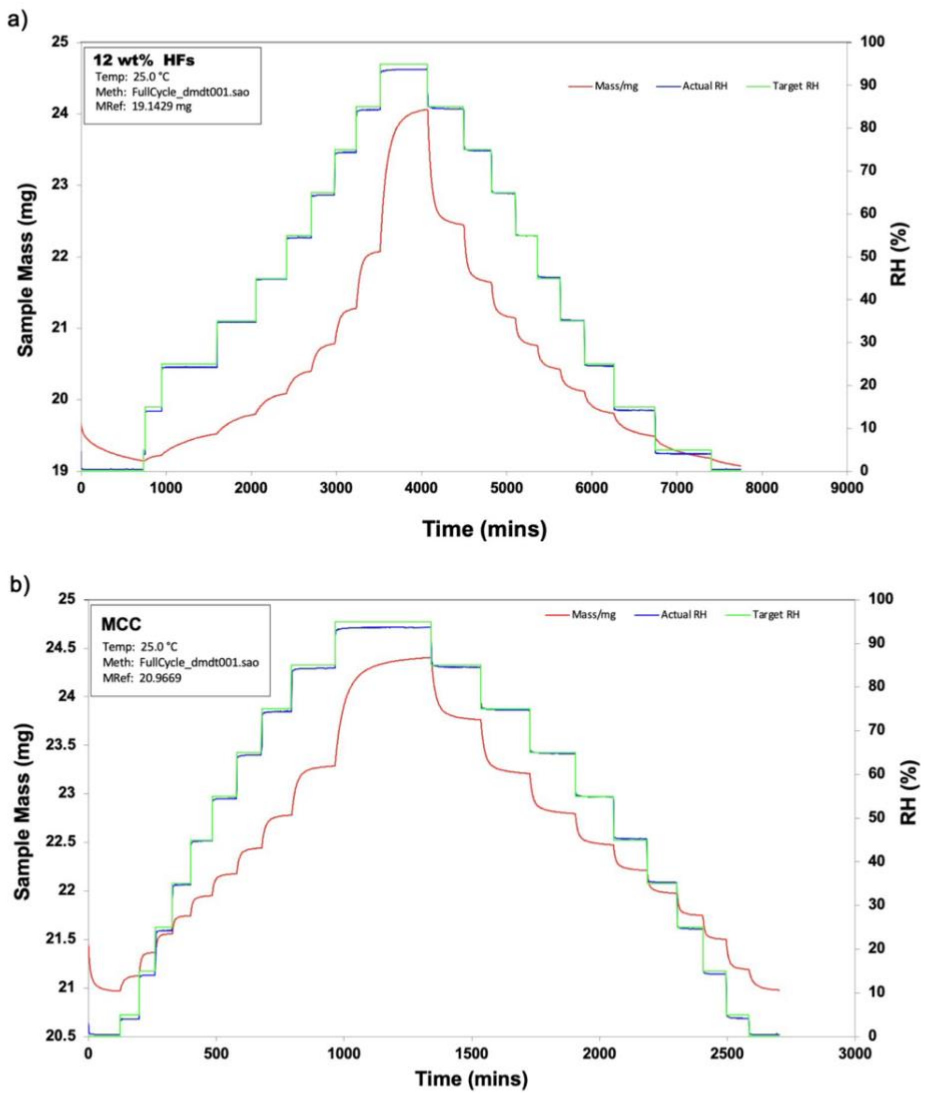

Figure S10. Sample weight of a.) HF and b.) MCC at actual RH as a function of time.

Table S6: Results of the equilibrium mass change (%) and hysteresis loop of the HF and MCC on adsorption and desorption against stepwise RH.

RH (%)	Adsorption Mass Changes (%)		Desorption Mass Changes (%)		Hysteresis (10%)	
-	HFs-12-6	MCC	HFs-12-6	MCC	HFs-12-6	MCC
0	0.00	0.00	-0.36	0.05	-	-
5	0.03	0.75	0.20	1.08	0.17	0.33
15	0.43	1.91	1.80	2.55	1.37	0.64
25	1.99	2.82	3.49	3.72	1.50	0.90

**35**	3.43	3.71	5.10	4.80	1.67	1.09
**45**	4.93	4.69	6.71	5.93	1.78	1.23
**55**	6.57	5.77	8.43	7.19	1.86	1.42
**65**	8.57	7.04	10.45	8.72	1.88	1.68
**75**	11.15	8.65	13.07	10.69	1.92	2.04
**85**	15.31	11.07	17.26	13.34	1.95	2.27
**95**	25.71	16.40	25.71	16.40	-	-

REFERENCES

(1) Michud, A.; Hummel, M.; Haward, S.; Sixta, H. Monitoring of Cellulose Depolymerization in 1-Ethyl-3-Methylimidazolium Acetate by Shear and Elongational Rheology. *Carbohydr. Polym.* **2015**, *117*, 355–363. DOI: 10.1016/j.carbpol.2014.09.075.

(2) Hummel, M.; Michud, A.; Ma, Y.; Roselli, A.; Stepan, A.; Hellstén, S.; Asaadi, S.; Sixta, H. High-Performance Lignocellulosic Fibers Spun from Ionic Liquid Solution. In *Cellulose Science and Technology*; John Wiley & Sons, Inc.: Hoboken, NJ, USA, 2018; pp 341–370.

(3) Hummel, M.; Michud, A.; Tanttu, M.; Asaadi, S.; Ma, Y.; Hauru, L. K. J.; Parviainen, A.; King, A. W. T.; Kilpeläinen, I.; Sixta, H. Ionic Liquids for the Production of Man-Made Cellulosic Fibers: Opportunities and Challenges. In *Advances in Polymer Science*; Springer International Publishing: Cham, 2015; pp 133–168.

(4) Lundahl, M. J.; Klar, V.; Wang, L.; Ago, M.; Rojas, O. J. Spinning of Cellulose Nanofibrils into Filaments: A Review. *Ind. Eng. Chem. Res.* **2017**, *56* (1), 8–19. DOI: 10.1021/acs.iecr.6b04010.

(5) Tan, X.; Chen, L.; Li, X.; Xie, F. Effect of Anti-Solvents on the Characteristics of Regenerated Cellulose from 1-Ethyl-3-Methylimidazolium Acetate Ionic Liquid. *Int. J. Biol. Macromol.* **2019**, *124*, 314–320. DOI: 10.1016/j.ijbiomac.2018.11.138.

(6) Lei, L.; Lindbråthen, A.; Sandru, M.; Gutierrez, M.; Zhang, X.; Hillestad, M.; He, X. Spinning Cellulose Hollow Fibers Using 1-Ethyl-3-Methylimidazolium Acetate–

Dimethylsulfoxide Co-Solvent. *Polymers (Basel)* **2018**, *10* (9), 972. DOI: 10.3390/polym10090972.

(7) Zhang, H.; Wu, J.; Zhang, J.; He, J. 1-Allyl-3-Methylimidazolium Chloride Room Temperature Ionic Liquid: A New and Powerful Nonderivatizing Solvent for Cellulose. *Macromolecules* **2005**, *38* (20),8272–8277. DOI: 10.1021/ma0505676.

(8) Nelson, M. L.; O'Connor, R. T. Relation of Certain Infrared Bands to Cellulose Crystallinity and Crystal Latticed Type. Part I. Spectra of Lattice Types I, II, III and of Amorphous Cellulose. *J. Appl. Polym. Sci.* **1964**, *8* (3), 1311–1324. DOI: 10.1002/app.1964.070080322.

(9) Falca, G.; Musteata, V.-E.; Behzad, A. R.; Chisca, S.; Nunes, S. P. Cellulose Hollow. Fibers for Organic Resistant Nanofiltration. J. Memb. Sci. 2019, 586, 151–161. DOI: 10.1016/j.memsci.2019.05.009.

(10) Li, H.-J.; Cao, Y.-M.; Qin, J.-J.; Jie, X.-M.; Wang, T.-H.; Liu, J.-H.; Yuan, Q. Development and Characterization of Anti-Fouling Cellulose Hollow Fiber UF Membranes for Oil–Water Separation. *J. Memb. Sci.* **2006**, *279* (1–2), 328–335. DOI: 10.1016/j.memsci.2005.12.025.

(11) Kim, D.; Vovusha, H.; Schwingenschlögl, U.; Nunes, S. P. Polyethersulfone Flat Sheet and Hollow Fiber Membranes from Solutions in Ionic Liquids. *J. Memb. Sci.* **2017**, *539*, 161–171. DOI: 10.1016/j.memsci.2017.06.001.

(12) Chu, Z.; Chen, K.; Xiao, C.; Ji, D.; Ling, H.; Li, M.; Liu, H. Improving Pressure. Durability and Fractionation Property via Reinforced PES Loose Nanofiltration Hollow Fiber Membranes for Textile Wastewater Treatment. J. *Taiwan Inst. Chem. Eng*. **2020**, *108*, 71–81. DOI: 10.1016/j.jtice.2019.12.009.

(13) Kim, D.; Le, N. L.; Nunes, S. P. The Effects of a Co-Solvent on Fabrication of. Cellulose Acetate Membranes from Solutions in 1-Ethyl-3-Methylimidazolium Acetate. *J. Memb. Sci.* **2016**, *520*, 540–549. DOI: 10.1016/j.memsci.2016.08.015.

(14) Ding, X.; Cao, Y.; Zhao, H.; Wang, L. Interfacial Morphology between the Two. Layers of the Dual-Layer Asymmetric Hollow Fiber Membranes Fabricated by Co-Extrusion and Dry-Jet Wet-Spinning Phase-Inversion Techniques. *J. Memb. Sci.* **2013**, *444*, 482–492. DOI: 10.1016/j.memsci.2013.03.035.

(15) Fan, Z.; Xiao, C.; Liu, H.; Huang, Q.; Zhao, J. Structure Design and Performance Study on Braid-Reinforced Cellulose Acetate Hollow Fiber Membranes. *J. Memb. Sci.* **2015**, *486*, 248–256. DOI: 10.1016/j.memsci.2015.03.066.
